# Adoptive Gay Father Families: Parent–Child Relationships and Children's Psychological Adjustment

**DOI:** 10.1111/cdev.12155

**Published:** 2013-08-22

**Authors:** Susan Golombok, Laura Mellish, Sarah Jennings, Polly Casey, Fiona Tasker, Michael E Lamb

**Affiliations:** University of Cambridge; Birkbeck University of London

## Abstract

Findings are presented on a U.K. study of 41 gay father families, 40 lesbian mother families, and 49 heterosexual parent families with an adopted child aged 3–9 years. Standardized interview and observational and questionnaire measures of parental well-being, quality of parent–child relationships, child adjustment, and child sex-typed behavior were administered to parents, children, and teachers. The findings indicated more positive parental well-being and parenting in gay father families compared to heterosexual parent families. Child externalizing problems were greater among children in heterosexual families. Family process variables, particularly parenting stress, rather than family type were found to be predictive of child externalizing problems. The findings contribute to theoretical understanding of the role of parental gender and parental sexual orientation in child development.

Research on the psychological development and well-being of children raised by same-sex parents has focused almost exclusively on families headed by lesbian mothers rather than gay fathers. Although it has consistently been shown that children with lesbian mothers do not differ from children in traditional families with respect to psychological adjustment or sex-typed behavior (Goldberg, 2010; Patterson, 2006, 2009), the circumstances of children with gay fathers are somewhat different. Not only are they raised by same-sex parents but also it is rare for fathers, whether heterosexual or gay, to be primary caregivers.

Research on fathering has shown that heterosexual fathers influence their children in similar ways to mothers (Lamb, 2010), but it remains the case that mothers are widely believed to be fundamentally more suited to parenting, for example, more nurturing, than are fathers (Biblarz & Stacey, 2010). Moreover, the wider social environment can have a marked impact on children's psychological well-being, and children with gay fathers may be exposed to greater prejudice and discrimination than children with lesbian mothers because gay father families possess the additional nontraditional feature of being headed by men (Golombok & Tasker, 2010). Regarding children's gender development, it is now generally agreed that sex-typed behavior results from an interplay among biological, psychological, and social mechanisms (Hines, 2010), with parents playing a minor, and possibly insignificant, role (Golombok & Tasker, 1996). Nevertheless, it has been suggested that the gender development of children with gay fathers may differ from that of children with lesbian mothers or heterosexual parents due to the presence of two male parents and the absence of a female parent from the home. Goldberg, Kashy, and Smith (2012) have postulated that children with gay fathers may show less sex-typed behavior than children with heterosexual parents resulting from a less sex-typed family environment, and girls in gay father families may show less sex-typed behavior than girls in lesbian mother families due to the absence of a female role model from the home.

In recent years, a growing number of gay father families have been created through adoption (Brodzinsky & Pertman, 2011). It has been estimated from the 2010 American Community Survey that around 7,100 adopted children are living with male couples (G. Gates, personal communication). Although adoption is associated with increased rates of adjustment problems for children in heterosexual families (Palacios & Brodzinsky, 2010), these appear to be largely related to factors that precede the adoption, such as abusive or neglectful parenting and multiple caretakers in the years before the adoption took place (Dozier & Rutter, 2008; Palacios & Brodzinsky, 2010). Nevertheless, adoption presents specific challenges for families (Grotevant & Von Korff, 2011) with poor communication about the adoption by adoptive parents, including the circumstances that led to the adoption and acknowledgment of the child's feelings about being adopted, associated with more negative psychological outcomes for children (Brodzinsky & Pinderhughes, 2002). In addition to the stressors experienced by adoptive parents generally, gay adoptive fathers may be exposed to stigma regarding their sexual identity (Goldberg, 2010). Those who are sensitive to such stigma have been found to show elevated levels of parenting stress (Tornello, Farr, & Patterson, 2011).

Initial investigations of adoptive gay father families have reported positive family functioning with respect to quality of parenting and children's psychological well-being (Averett, Nalavany, & Ryan, 2009; Erich, Kanenberg, Case, Allen, & Bogdanos, 2009; Erich, Leung, & Kindle, 2005; Leung, Erich, & Kanenberg, 2005; Ryan, 2007). However, reliance on self-report questionnaires administered to convenience samples, and either the absence of a comparison group of heterosexual adoptive families or the wide age range of children studied, limit the conclusions that may be drawn. The first systematic study was carried out by Farr, Forsell, and Patterson (2010a, 2010b). Using parent and teacher questionnaires, preschool children adopted in infancy by gay fathers in the United States were found to be as well adjusted as those adopted by lesbian or heterosexual parents, with no differences in parenting stress, parental discipline, or parental relationship satisfaction according to family type. In terms of gender development, no differences were identified in the sex-typed behavior of either boys or girls between gay father, lesbian mother, and heterosexual parent families. In contrast, Goldberg et al. (2012) found that children in adoptive same-sex parent families showed less sex-typed behavior than children in heterosexual parent families. This appeared to reflect less masculine play by boys in lesbian mother families rather than less feminine play by girls with gay fathers. This study contributes to this emerging body of research by investigating larger samples of gay, lesbian, and heterosexual adoptive families in the United Kingdom using standardized interview and observational and questionnaire measures of parental well-being, quality of parent–child relationships, child adjustment, and child sex-typed behavior, administered to parents, children, and teachers.

From a theoretical perspective, the study is founded upon a developmental systems approach (Lerner, Lewin-Bizan, & Warren, 2011), whereby bidirectional relations between individuals, the family, and the wider social world, including historical time and place, are viewed as influential in development. More specifically, the study was guided by the theoretical and research literature on parenting that shows that the quality of children's relationships with their parents, including warmth, sensitivity, and appropriate discipline and control, as well as parental psychological well-being, is associated with positive child adjustment (Bornstein, 2002; Collins, Maccoby, Steinberg, Hetherington, & Bornstein, 2000; Lamb, 2012). Despite the commonly held assumption that gay fathers may be less nurturing than lesbian or heterosexual mothers, and the possibility that they may be exposed to greater prejudice, existing research suggests that gay father families would not differ from lesbian or heterosexual families with respect to parenting processes such as warmth and sensitivity that are associated with children's psychological adjustment. However, studies in the United States provide some indication that adoption agencies tend to place children from the most difficult backgrounds and with the most challenging behaviors with same-sex parents (Brodzinsky & Evan B. Donaldson Adoption Institute, 2011; Brooks & Goldberg, 2001; Matthews & Cramer, 2006). If this is similarly the case in the United Kingdom, less positive parenting and child adjustment may be predicted for gay father families. It was also hypothesized, based on the growing body of research showing that family structure is less predictive of child adjustment than the quality of parent–child relationships (Biblarz & Stacey, 2010; Golombok, 2000, 2013; Lansford, Ceballo, Abbey, & Stewart, 2001; Patterson, 2006, 2009), that parenting processes would be more strongly associated with child adjustment than family type.

This is the first study of adoptive gay and lesbian families to be conducted outside the United States and is of particular interest as a change in legislation in the United Kingdom that came into force in 2005 has enabled gay and lesbian couples to become joint legal parents of their adopted children. Unlike the United States, where intercountry adoption and interracial adoption are common (Russett, 2012), more than 95% of children adopted in the United Kingdom are adopted from the child welfare system and interracial adoption is strongly discouraged. Furthermore, private adoption is not allowed. The average time between a child entering public care and being placed with an adoptive family is 21 months (Department for Education, 2012).

## Method

### Participants

Forty-one two-parent gay adoptive families, 40 two-parent lesbian adoptive families, and 49 two-parent heterosexual adoptive families participated in the study. With the aid of the British Association of Adoption and Fostering, adoption agencies that had placed children with same-sex parents assisted with recruitment by contacting gay, lesbian, and heterosexual adoptive parents who had adopted children through their agency. In addition, two support groups for gay and lesbian adoptive families sent information about the research to their members. The inclusion criteria were that the target child was aged between 4 and 8 years and had been placed with the adoptive family for at least 12 months. Two children within 1 month of reaching age 4 years and two children who had just passed their 9th birthday were included to maximize sample size. Not all the agencies involved in recruitment kept systematic records of the families they had contacted. However, for those that did so, a participation rate of 71% was obtained.

As shown in Table 1, there was no difference between family types in the age of the target child, with the average age being 6 years. However, a significant difference was found with respect to the children's gender, χ^2^(2) = 12.74, *p* = .002. Whereas the heterosexual families had an equal number of boys and girls, there was a preponderance of boys adopted by gay fathers and a preponderance of girls adopted by lesbian mothers. There was also a difference between family types in the age of the child at adoption, *F*(2, 127) = 4.82, *p* = .01, and the length of placement with the adoptive family, *F*(2, 127) = 4.08, *p* = .02, with children in gay father families being older at the time of adoption and placed for a shorter period of time. The number of siblings in the adoptive families did not differ according to family type, with the large majority of children having a maximum of one sibling. All but one child was attending nursery or school. There was no difference between family types in the number of hours per week children spent in nonparental care.

**Table 1:** Means, Standard Deviation, *F*, *p,* and *d* Values for Sociodemographic Information by Family Type

	Gay (G)		Lesbian (L)		Heterosexual (H)				G vs. L	G vs. H
	*M*	*SD*	*M*	*SD*	*M*	*SD*	*F*	*p*	*d*	*d*
Age of child (months)	73.12	17.52	75.82	21.35	72.00	16.60	0.48	.61	.14	.07
Age of child at adoption	39.88	19.93	35.85	23.49	26.71	18.87	4.82	.01	.19	.68
Length of placement	32.98	18.34	40.03	21.63	45.27	20.77	4.08	.02	.35	.62
Nonparental care (hours per week)	6.83	4.74	5.94	6.90	5.05	3.88	2.70	.11	.15	.41
Age of Parent A	40.37	5.73	42.43	7.28	43.16	5.32	2.44	.09	.31	.05
Age of Parent B	40.07	4.72	43.15	6.97	43.67	5.56	4.81	.01	.52	.69

	*N*	%	*N*	%	*N*	%	χ^2^	*p*	Kramer's *V*	
Child's sex										
Male	32	78.0	16	40.0	25	51.0	12.74	.002	.39	.28
Female	9	22.0	24	60.0	24	49.0				
No. of preadoptive placements										
None/one	25	61.0	22	55.0	35	71.4	5.14	.27	.11	.14
Two	12	29.2	11	27.5	12	24.5				
Three or more	4	9.8	7	17.5	2	4.1				
Siblings										
None	12	29.2	13	32.5	10	20.4	4.40	.35	.13	.19
One	20	48.8	22	55.0	33	67.3				
Two or more	9	22.0	5	12.5	6	12.2				
Parent A working status										
Not working	11	26.8	8	20.0	16	32.7	8.89	.06	.11	.29
Part time	15	36.6	19	47.5	27	55.1				
Full time	15	36.6	13	32.5	6	12.2				
Parent B working status										
Not working	1	2.4	2	5.0	43	4.1	6.65	.15	.22	.05
Part time	4	9.8	10	25.0	4	8.2				
Full time	36	87.8	28	70.0	2	87.8				
Parent A occupation										
Professional/managerial	26	86.7	26	81.2	22	66.7	3.96	.14	.07	.23
Skilled/partly skilled	4	13.3	6		11					
Parent B occupation										
Professional/managerial	35	89.7	32	82.1	33	70.2	5.23	.07	.11	.24
Skilled/partly skilled	4	10.3	7	17.9	14	29.8				
Parent A ethnic identity										
White	39	95.1	35	87.5	49	100	6.78	.03	.14	.16
Other	2	4.9	5	12.5	0	0				
Parent B ethnic identity										
White	38	92.2	38	95.0	45	91.8	.35	.83	.05	.02
Other	3	7.3	2	5.0	4	8.2				
Relationship status										
Married/civil partnership	30	73.2	31	77.5	46	93.9	7.49	.02	.05	.28
Unmarried/no civil partnership	11	26.8	9	22.5	3	6.1				

It was not possible to collect comprehensive data on the children's preadoption history as not all the parents had accurate information on their child's early experiences. For gay, lesbian, and heterosexual families, respectively, where information was available, the proportion of birth mothers with mental health problems was 34.1%, 32.5%, and 38.8% (19.2% not known); the proportion who had experienced domestic violence was 31.7%, 45.0%, and 40.8% (21.5% not known); the proportion who had abused alcohol was 36.6%, 55.0%, and 36.7% (20% not known); and the proportion of birth fathers who had been convicted of criminal behavior was 39.0%, 35.0%, and 32.7% (36.2% not known). For children in gay, lesbian, and heterosexual adoptive families, respectively, where information was available, the proportion who had experienced neglect was 78.0%, 62.5%, and 59.2% (3.1% not known); the proportion who had experienced emotional abuse was 46.3%, 45.0%, and 30.6% (8.2% not known); and the proportion who had experienced physical abuse was 14.6%, 20.0%, and 14.3% (10.2% not known). There were no significant differences between family types in the proportion of children who had experienced each of these adversities. Regarding contact with the birth family, 59.5%, 52.9%, and 50.0% of children in gay, lesbian, and heterosexual parent families, respectively, had no contact, with no significant difference between family types, and the large majority of those with contact exchanged letters only, once or twice per year.

For presenting demographic data for each parent individually, the parent who was most involved with the child on a day-to-day basis according to parent reports, and agreed by two interviewers (LM and SJ), was labeled Parent A and the coparent was labeled Parent B, in the lesbian and gay families. Although the parents generally shared child care, Parent B usually spent more time in employment and slightly less time with the child. In the small number of families where parents shared child care evenly, Parent A and Parent B were assigned randomly. In the heterosexual families the mother was Parent A and the father was Parent B. There was no difference between family types in the age of Parent A. However, the age of Parent B differed significantly between groups, *F*(2, 127) = 4.81, *p* = .01, reflecting the younger age of Parent B in gay father families. The working status of Parent A and Parent B did not differ according to family type. Social class was assessed according to a modified version of the U.K. Registrar General's classification (OPCS & Employment Department Group, 1991). Each parent's occupation was classified as either professional/managerial or skilled/partly skilled. The family types did not differ with respect to parental occupation, with the majority of parents employed in professional or managerial positions. Whereas there was no group difference in the ethnic identity of Parent B, there was greater diversity in the ethnic identity of Parent A in lesbian and gay families, χ^2^(2) = 6.78, *p* = .03. There was also a difference in relationship status between family types, χ^2^(2) = 7.49, *p* = .02, reflecting a lower proportion of gay and lesbian parents in civil partnerships than of heterosexual parents who were married.

### Procedure

The families were assessed at home. Written informed consent to participate in the investigation was obtained from each parent and verbal assent was obtained from the child. Ethical approval was granted by the University of Cambridge Psychology Research Ethics Committee. Each parent was administered an audio-recorded standardized interview that lasted approximately 1.5 hr and standardized questionnaires, and participated in a video-recorded observational task with the child that lasted 5–10 min. Teachers completed a questionnaire designed to assess the children's psychological adjustment. Written informed consent was obtained from teachers. To provide interrater reliability ratings for the interview and observational measures, data from 40 randomly selected families were coded by a second interviewer who was blind to family type.

### Measures

#### Parenting

##### Questionnaires of parental well-being

The Trait Anxiety Inventory (Spielberger, 1983), the Edinburgh Depression Scale (Thorpe, 1993), and the short form of the Parenting Stress Index (PSI/SF; Abidin, 1990) were completed by each parent to assess anxiety, depression, and stress associated with parenting, respectively. Each of these instruments, for which higher scores represent greater difficulties, has been shown to have good reliability and to discriminate well between clinical and nonclinical groups. Cronbach's alpha for the Trait Anxiety Inventory, the Edinburgh Depression Scale, and the PSI, respectively, in this study was 0.91, 0.81, and 0.92.

##### Interview with parents

Each parent was interviewed separately using an adaptation of a semistructured interview designed to assess quality of parenting that has been validated against observational ratings of mother–child relationships in the home (Quinton & Rutter, 1988). Detailed accounts are obtained of the child's behavior and the parent's response to it, with particular reference to interactions relating to warmth and control. A flexible style of questioning is used to elicit sufficient information for each variable to be rated by the researcher using a standardized coding scheme.

The following variables were coded: (a) *expressed warmth* from 0 (*none*) to 5 (*high*) took account of the parent's tone of voice, facial expressions, and gestures, in addition to what the parent said about the child; (b) *sensitive responding* from 0 (*none*) to 4 (*high*) represented the parent's ability to recognize and respond appropriately to the child's needs; (c) *enjoyment of play* from 1 (*little or none*) to 4 (*a great deal*) assessed the extent to which the parent and child engaged in joint activities and enjoyed each other's company; (d) *amount of interaction* from 1 (*a little*) to 3 (*high*) assessed the amount of time the parent and child spent in shared activities; (e) *quality of interaction* from 1 (*poor*) to 4 (*very good*) was based on the extent to which the parent and child wanted to be with each other and showed each other affection; (f) *frequency of battle* from 0 (*never/rarely*) to 5 (*few times daily*) assessed the frequency of parent–child conflict; (g) *level of battle* from 0 (*none*) to 3 (*major*) assessed the severity of parent–child conflict; (h) *disciplinary indulgence* from 0 (*none*) to 4 (*somewhat indulgent*) was based on the extent to which the parent let the child get away with things; and (i) *disciplinary aggression* from 0 (*none*) to 3 (*somewhat aggressive*) was based on the level of anger shown by the parent toward the child. The interrater reliabilities (intraclass correlation coefficients) were as follows: expressed warmth (.75), sensitive responding (.71), enjoyment of play (.87), amount of interaction (.82), quality of interaction (.77), frequency of battles (.95), level of battles (.85), disciplinary indulgence (.74), and disciplinary aggression (.76).

##### Parent–child observations

The Etch-A-Sketch task (Stevenson-Hinde & Shouldice, 1995) was used to obtain an observational assessment of interaction between the primary parent and the child. The Etch-A-Sketch is a drawing tool with two dials that allow one person to draw vertically and the other to draw horizontally. The parent and child were asked to copy a picture of a house, each using one dial only, with clear instructions not to use the other dial. The Coconstruction task (Steele et al., 2007) was used to obtain an observational assessment of parent–child interaction with the coparent. The parent and child were given a set of wooden building blocks and instructed to build something together using as many blocks as possible. The sessions were video recorded and coded using the Parent–Child Interaction System (Deater-Deckard & Petrill, 2004) to assess the construct of mutuality, that is, the extent to which the parent and child engaged in positive dyadic interaction characterized by warmth, mutual responsiveness, and cooperation. The following variables were rated on a 7-point scale ranging from 1 (*no instances*) to 7 (*constant, throughout interaction*): (a) *child's responsiveness to parent* assessed the extent to which the child responded immediately and contingently to the parent's comments, questions, or behaviors; (b) *parent's responsiveness to child* assessed the extent to which the parent responded immediately and contingently to the child's comments, questions, or behaviors; (c) *dyadic reciprocity* assessed the degree to which the dyad showed shared positive affect, eye contact, and a “turn-taking” (conversation like) quality of interaction; and (d) *dyadic cooperation* assessed the degree of agreement about whether and how to proceed with the task. The interrater reliabilities (intraclass correlation coefficients) were as follows: .65 for child responsiveness, .52 for parent responsiveness, .83 for dyadic reciprocity, and .75 for dyadic cooperation.

#### Child Adjustment

##### Strengths and Difficulties Questionnaire

The presence of child psychological problems was assessed with the Strengths and Difficulties Questionnaire (SDQ; Goodman, 1994, 1997) administered to the primary parent and the child's teacher to produce scores of externalizing problems and internalizing problems (Goodman, Lamping, & Ploubidis, 2010), with higher scores indicating greater problems. The SDQ has been shown to have good internal consistency, test–retest and interrater reliability, and concurrent and discriminative validity (Goodman, 1994, 1997, 2001; Stone, Otten, Engels, Vermulst, & Janssens, 2010). The number of children obtaining a parent-rated total SDQ score above cutoff for psychiatric disorder was also calculated.

##### Sex-typed behavior

Sex-typed behavior was assessed using the Preschool Activities Inventory (PSAI), a psychometrically constructed questionnaire designed to differentiate within as well as between the sexes, with higher scores representing more male-typical behavior (Golombok et al., 2008). This questionnaire was mailed to parents for completion following the home visit. The PSAI has been standardized on more than 2,000 children in the United Kingdom, the Netherlands, and the United States (Golombok & Rust, 1993a). Split-half reliability is .66 for boys and .80 for girls, and test–retest reliability over a 1-year period is .62 for boys and .66 for girls. The inventory has been validated on boys and girls attending day care in five different centers. Significant correlations were found between mothers and teachers' ratings of gender-typed behavior, showing the inventory to be a valid measure of gender role (Golombok & Rust, 1993a, 1993b).

## Results

### Analysis Plan

As data on parenting were collected from both parents, which produces a clustered data structure, multilevel regression models were estimated to compare family types on the following constructs: parents' psychological well-being (Trait Anxiety Inventory, Edinburgh Depression Scale, and PSI), warmth (expressed warmth and sensitive responding), interaction (enjoyment of play, amount of interaction, and quality of interaction), conflict (frequency of battle, level of battle, disciplinary indulgence, and disciplinary aggression), and the observational variables relating to mutuality (child responsiveness, parent responsiveness, dyadic reciprocity, and dyadic cooperation). Family-level covariates included the child's age at adoption and length of placement with the adoptive family. A robust maximum likelihood estimator was used to address the nonnormality in the data. The measure of child adjustment, the SDQ, was administered to one parent only and to the child's teacher. Multivariate analyses of covariance (MANCOVAs), with the child's age at adoption and length of placement with the adoptive family entered as covariates, were conducted separately for externalizing problems and internalizing problems for both parents' and teachers' data. Similarly, the PSAI was administered to one parent, and a MANCOVA was conducted with family type and gender of the child as between-subjects factors, and the child's age at adoption and length of placement with the adoptive family entered as covariates. The analyses focused on the following comparisons to address specific questions: (a) *gay* versus *lesbian* to examine whether families headed by male same-sex parents differed from families headed by female same-sex parents controlling for adoption and (b) *gay* versus *heterosexual* to examine whether families headed by male same-sex parents differed from traditional heterosexual families controlling for adoption. To examine whether family structure or the family process variables played a more important role in the prediction of child adjustment, a single-level multiple regression model was estimated for the parenting variables that differed between family types, the child variables that differed between family types, and family type. As only one parent completed the child adjustment questionnaire, parenting data from that parent only was used in the analysis. Standardized results based on robust maximum likelihood estimation are presented.

### Parenting

As shown in Table 2, significant differences between gay and heterosexual families were found for parents' psychological well-being. Specifically, gay fathers showed lower levels of both depression (*b* = 1.57, *p* = .008) and parenting stress (*b* = 10.62, *p* = .003) in comparison to heterosexual mothers and fathers. With respect to parenting quality, a difference between family types was found for warmth, with higher levels of expressed warmth shown by gay than heterosexual parents (*b* = −.26, *p* = .04). In addition, gay parents were found to interact more with their children than did heterosexual parents (*b* = −.20, *p* = .01). For the observational assessment of parent–child interaction, a significant difference between gay and heterosexual parent families was found for parent responsiveness (*b* = −.22, *p* = .007), reflecting greater responsiveness by gay than heterosexual parents. Disciplinary aggression also differed significantly between family types, with lower levels of disciplinary aggression shown by gay than heterosexual parents (*b* = .30, *p* = .005). The comparisons between gay and lesbian families showed no significant differences between family types.

**Table 2:** Means, Standard Deviation (*M*), β, *p*, and *d* Values for Parents' Psychological Well-Being, Warmth, Interaction, Conflict, and Mutuality by Family Type

	Gay (G)		Lesbian (L)		Heterosexual (H)		G vs. L				G vs. H			
	*M*	*SD*	*M*	*SD*	*M*	*SD*	*b*	*SE*	*p*	*d*^a^	*b*	*SE*	*p*	*d*^b^
Psychological well-being														
Trait Anxiety Inventory	34.20	8.00	35.68	8.95	36.70	8.98	1.12	1.51	.45	.17	2.23	1.50	.13	.29
Edinburgh Depression Scale	4.46	3.08	5.68	3.93	5.94	4.10	1.16	.63	.06	.35	1.57	.59	.008	.40
Parenting Stress Index	66.81	17.21	69.68	18.55	75.86	19.85	2.80	3.36	.40	.16	10.62	3.62	.003	.49
Warmth														
Expressed warmth	3.88	.78	3.79	.92	3.66	.76	−.08	.14	.56	.11	−.26	.13	.04	.28
Sensitive responding	2.91	.63	2.83	.76	2.69	.66	−.08	.12	.48	.11	−.21	.11	.06	.34
Interaction														
Enjoyment of play	3.33	.63	3.25	.85	3.14	.77	−.04	.12	.75	.11	−.16	.11	.13	.27
Amount of interaction	2.56	.50	2.53	.59	2.35	.58	−.01	.08	.95	.05	−.20	.08	.01	.38
Quality of interaction	3.32	.59	3.28	.66	3.13	.62	−.02	.10	.83	.06	−.19	.10	.07	.31
Conflict														
Frequency of battle	3.12	1.19	3.01	1.13	3.48	1.21	−.15	.21	.48	−.09	−.15	.21	.32	.30
Level of battle	1.56	.74	1.80	.79	1.65	.77	.21	.14	.13	.31	.09	.14	.52	.12
Disciplinary indulgence	1.39	.78	1.38	.66	1.64	.66	−.04	.13	.75	−.01	.16	.13	.20	.35
Disciplinary aggression	1.09	.63	1.34	.69	1.38	.63	.22	.12	.06	.38	.30	.10	.005	.46
Mutuality														
Child responsiveness	5.74	.64	5.76	.74	5.65	.95	.01	.11	.91	−.03	−.07	.13	.54	.11
Parent responsiveness	6.47	.58	6.34	.66	6.28	.64	−.12	.08	.17	.21	−.22	.08	.007	.31
Dyadic reciprocity	2.14	.90	2.29	.99	2.24	.93	.14	.14	.35	−.16	.11	.82	.41	−.11
Dyadic cooperation	2.66	.95	2.80	.97	2.65	1.02	.16	.15	.28	−.15	.07	.15	.63	.01

a Positive *d* values represent more positive outcome for gay father than lesbian mother families.

b Positive *d* values represent more positive outcome for gay father than heterosexual parent families.

### Child Adjustment

MANCOVAs were carried out separately for the subscales of the SDQ relating to externalizing problems (conduct problems and hyperactivity) and internalizing problems (emotional problems and peer problems). Wilks's lambda was significant for externalizing problems, *F*(4, 246) = 2.74, *p* = .03. One-way ANCOVAs identified a significant difference between groups for hyperactivity, *F*(2, 124) = 4.20, *p* = .02. Children in heterosexual families showed the highest levels of hyperactivity (see Table 3). Teachers' externalizing and internalizing subscale scores were entered into separate MANCOVAs. Wilks's lambda was not significant. There was no difference between family types in the proportion of children who obtained parent-rated total SDQ scores above cutoff for psychiatric disorder (14.6%, 12.8%, and 18.4% of children in gay father, lesbian mother, and heterosexual parent families, respectively).

**Table 3:** Means, Standard Deviation, *F*, *p*, and *d* Values for Child Adjustment by Family Type

	Gay (G)		Lesbian (L)		Heterosexual (H)				G vs. L	G vs. H
	*M*	*SD*	*M*	*SD*	*M*	*SD*	*F*	*p*	*d*^a^	*d*^b^
Parent Strengths and Difficulties Questionnaire (SDQ)										
Externalizing problems							2.74	.03		
Conduct problems	2.12	2.10	2.33	2.08	2.90	1.80	2.66	.07	.10	.40
Hyperactivity	4.61	2.40	4.05	2.50	5.18	2.79	4.20	.02	−.23	.22
Internalizing problems							1.02	.40		
Emotional problems	1.83	2.49	1.62	1.91	1.98	1.77	.74	.47	−.09	.07
Peer problems	1.61	1.73	1.92	1.70	1.51	1.68	.57	.57	.18	−.06
Teacher SDQ										
Externalizing problems							.41	.79		
Conduct problems	1.62	2.26	1.33	1.62	1.82	2.38	.45	.64	−.15	.08
Hyperactivity	4.75	2.79	4.37	2.97	4.95	3.31	.70	.50	−.13	.06
Internalizing problems							1.55	.19		
Emotional problems	1.34	1.53	1.83	2.08	2.05	2.36	1.01	.37	.27	.34
Peer problems	1.78	2.53	1.17	1.48	2.12	1.76	2.12	.12	−.29	.16

a Positive *d* values represent more positive outcome for gay father than lesbian mother families.

b Positive *d* values represent more positive outcome for gay father than heterosexual parent families.

With respect to sex-typed behavior, a MANCOVA with family type and gender of the child as between-subjects factors was conducted for PSAI scores. Although, as expected, there was a significant difference according to child's gender, *F*(1, 115) = 202.72, *p* < .0001, there was no significant difference for family type or for the interaction between family type and child's gender.

### Parenting, Family Type, and Child Adjustment

To examine the relative importance of the family process variables and family type in predicting child adjustment, the parenting variables (depression, parenting stress, expressed warmth, amount of interaction, disciplinary aggression, and parent responsiveness) and the child variables (age at adoption and length of placement) that differed between family types, as well as family type, were entered into a multiple regression analysis. The dependent variable was child externalizing problems as assessed by parents, as this variable differed according to family type. Parenting stress was found to predict child externalizing problems (β = .58, *p* < .001), with higher levels of parenting stress associated with higher levels of child externalizing problems. In addition, disciplinary aggression was marginally predictive of child externalizing problems (β = .12, *p* = .07). Family type did not predict child externalizing problems.

## Discussion

Where differences were identified between the gay father adoptive families and the heterosexual parent adoptive families, these reflected more positive functioning in the gay father families. Regarding the psychological well-being of the parents, the gay fathers showed lower levels of depression and stress associated with parenting than the heterosexual parents. There were no differences in psychological well-being between the gay fathers and the lesbian mothers. The positive findings for psychological well-being are consistent with those of Goldberg and colleagues (Goldberg & Smith, 2011; Goldberg, Smith, & Kashy, 2010) in relation to gay and lesbian couples across the 1st year of adoptive parenthood, and Farr et al. (2010b) and Tornello et al. (2011) with respect to gay and lesbian adoptive parents with a preschool or early school-age child, who found similar parental mental health outcomes across lesbian mother, gay father, and heterosexual parent families.

In terms of parenting, gay fathers showed higher levels of warmth, greater amounts of interaction, and lower levels of disciplinary aggression as assessed by interview, as well as higher levels of responsiveness as assessed by direct observation, than the heterosexual parents. No differences in parenting were identified between gay fathers and lesbian mothers. With respect to child adjustment, externalizing problems as rated by parents were greater among children in heterosexual than in gay and lesbian families. As expected with an adoptive sample, 15.5% of children obtained parent-rated total SDQ scores above cutoff for psychiatric disorder in comparison to the 8% reported in U.K. general population norms (Meltzer, Gatward, Goodman, & Ford, 2000). There was no difference in the proportion of children obtaining scores above cutoff between family types.

The more positive outcomes for gay father families in terms of parental well-being and parent–child relationships may be associated with characteristics of the parents or of the children. Adoption by gay couples is a relatively new phenomenon in the United Kingdom that has attracted much controversy (Hill, 2009). It seems likely, therefore, that the screening process is especially stringent for gay couples who wish to adopt, resulting in even higher levels of psychological well-being and commitment to parenting among adoptive gay fathers than adoptive lesbian or heterosexual parents. Moreover, unlike heterosexual couples, it may be relevant that gay fathers have not experienced the stress of infertility and failed fertility treatments, and have not turned to adoption as a second choice in their quest for a child.

It is also conceivable, due to concerns regarding adoption by gay men, that children with higher levels of psychological problems were least likely to be placed with gay couples. The lower levels of child externalizing problems among children with gay fathers suggest that this may be the case. However, children adopted by gay fathers had been adopted at an older age and placed with the adoptive family for a shorter time, both of which have been associated with greater adjustment problems (Dozier & Rutter, 2008; Palacios & Brodzinsky, 2010). Moreover, from the available data on children's preadoption history, it appeared that those placed with gay fathers were no less likely to have experienced serious adversity such as neglect, or emotional or physical abuse, than children placed with lesbian mothers or heterosexual parents. Neither were their birth mothers less likely to have experienced mental health problems, domestic violence, or alcohol abuse, nor were their fathers less likely to have been convicted of criminal behavior. Although research in the United States provides some indication that the most difficult children may be placed with same-sex parents (Brodzinsky & Evan B. Donaldson Adoption Institute, 2011; Brooks & Goldberg, 2001; Matthews & Cramer, 2006), this does not currently appear to be the case in the United Kingdom, perhaps because almost all adoptions involve children who have experienced adversity in their early years. It appears, therefore, that rather than adopting less difficult children, gay fathers provide a highly positive parenting environment for their adopted children, although, given the bidirectional nature of parent–child relationships, both factors are likely to be at play.

The findings of this study, conducted in the United Kingdom, contribute to the small amount of existing data on parenting and child development in adoptive gay father families, and support the conclusions of Farr et al. (2010a) in the United States that gay men make capable adoptive parents. Whereas Farr et al. (2010a) reported no differences in parenting or child adjustment between adoptive gay fathers and either adoptive lesbian mothers or adoptive heterosexual parents, the differences identified in this study reflected more positive parenting and child adjustment in gay father families. This discrepancy may result from the larger sample in this study, or the use of more in-depth measures of the quality of parent–child relationships such as standardized interviews involving detailed questioning and the assessment of nonverbal aspects of the parents' responses, as well as observational assessments of the quality of interactions between parents and their children. This study focused on an older sample of children who had experienced early adversity before being placed with their adoptive parents, showing that gay fathers cope well with the challenges posed by children from difficult backgrounds. It is interesting to note that this study, as well as the earlier studies by both Farr et al. (2010a) and Brodzinsky (2011), found that gay fathers were more likely to have adopted boys, whereas lesbian mothers were more likely to have adopted girls. It is not known whether this resulted from a tendency for adoption agencies to gender-match children to same-sex parents, or from same-sex parents' preference for a child of the same gender as themselves. Anecdotal evidence from the present investigation suggests that same-sex parents tended not to express a preference regarding the gender of the child.

The findings are also of more general theoretical interest regarding the influence of parenting on child development. Much of the limited previous research on fathers as primary parents has focused on single fathers (e.g., Santrock, Warshak, & Elliott, 1982) or on fathers who assume primary responsibility for limited periods of time (Russell, 1999). Comparisons between gay and lesbian families enable the influence of parental gender on child development to be examined in a novel way by controlling for the presence of two parents. Although such “natural experiments” are not free of methodological problems, they are informative in that they allow the separation of factors that in traditional families occur together (Rutter, 2007; Rutter, Pickles, Murray, & Eaves, 2001). The findings of this study suggest that men can be just as competent at parenting as women, and that the absence of a female parent does not necessarily have adverse consequences for child adjustment. Moreover, the finding that externalizing problems in children are associated with high levels of parenting stress but not family type, replicates that of Farr et al. (2010a, 2010b) with a sample from a different geographical area, and adds weight to the growing body of evidence that family processes are more influential in child adjustment than is family structure (Biblarz & Stacey, 2010; Golombok, 2000, 2013; Lansford et al., 2001; Patterson, 2006, 2009).

The lack of difference in sex-typed behavior between children with gay fathers and children with lesbian or heterosexual parents for either boys or girls is consistent with previous research on young children with same-sex parents, and supports the conclusion that parental sexual orientation has little influence on the gender development of young children (Golombok et al., 2003; Patterson, 2006, 2009). Two studies have specifically investigated the sex-typed behavior of children adopted by gay fathers. The findings of Farr et al. (2010a) were similar to those of the present investigation in that no differences were identified according to family type for either boys or girls. However, Goldberg et al. (2012) found less sex-typed behavior among children of same-sex parents. This appeared to reflect less masculine play behavior among the sons of lesbian mothers rather than less feminine play behavior among the daughters of gay fathers, and may result from the younger age of the children under study. This issue warrants further investigation.

The study had a number of limitations. Differences between family types may not have been detected due to the modest sample sizes. For the comparisons between the gay father and heterosexual parent families, and between the gay father and lesbian mother families, the smallest *d* (standardized difference between means) that could be detected as statistically significant was around 0.30 and .32, respectively, for a power of 0.80. Thus, to the extent that significant differences between family types were not identified due to insufficient power, these differences would have been small. Moreover, Type II errors may have resulted from the moderate interrater reliability of some variables. However, the coding of the interview variables involved the use of nonverbal cues such as facial expression and gestures that were not available to the second rater. Thus, the interrater reliabilities of the interview variables were may be underestimated. For the observational assessment, the reliability of the parent responsiveness variable was low. Inspection of the data showed that this was due to ceiling effects in these highly functioning families; most families obtained a score in the top 2 points of the scale. This variable has been shown to be reliable in studies of more diverse samples (Deater-Deckard & Petrill, 2004), including studies conducted by our own research group (Ensor & Hughes, 2009; Golombok et al., 2011). Thus, rather than being unreliable in terms of detecting low parent responsiveness, this rating appears to be less reliable when discriminating between scores at the upper (high responsiveness) end of the scale. As the SDQ and the PSAI were administered to one parent only, it was not possible to conduct multilevel analyses with these variables. A further limitation was that not all the adoption agencies involved in recruitment kept systematic records of the families they had contacted. However, for those that did so, a participation rate of 71% was obtained. National statistics show that approximately 60 children are adopted by gay couples and 60 by lesbian couples in the United Kingdom each year (Department for Education, 2010). As these figures apply to children of all ages, it appears that our samples of 41 gay father and 40 lesbian mother families comprise a large proportion of the eligible gay father and lesbian mother families in the United Kingdom with an adopted child aged 4–8 years.

An advantage of the study was the multimethod (interview, observation, and questionnaire), multiinformant (both parents, child, and teacher) design, as gay parents, in particular, may tend to present their families as high functioning, either in response to the stigma they experience in the outside world or because they feel they must live up to high expectations of themselves as parents given the difficulties they faced in adopting children. The observational measures are especially useful in this regard as it is difficult to “fake good” with observational measures (Kerig, 2001) that provide an assessment of the quality of dynamic interactions between parents and their children that cannot be captured by interview or self-report (Aspland & Gardner, 2003; Bakeman & Gottman, 1997; Hartmann & Wood, 1990). Although not all the teachers participated, completed questionnaires were returned by 78%, with no difference in the proportion of missing teachers' questionnaires between family types. In addition, there was no difference in parents' SDQ scores between families with and without teacher questionnaires.

The findings of this study have implications for the development of policy and legislation in relation to the creation of gay father families through adoption. At a time when there are many children waiting to be adopted, but a shortage of suitable adopters, the positive findings regarding the gay adoptive families in this study suggest that there exists a largely untapped pool of potential adoptive parents. The challenges faced by gay couples who wish to adopt are even greater than those experienced by lesbian and heterosexual couples. It seems that those who successfully complete the adoption process become particularly committed parents.
